# Percutaneous Closure of Patent Foramen Ovale in a Patient With Incomplete Cor Triatriatum Sinister: A Case Report

**DOI:** 10.1002/jcu.70253

**Published:** 2026-04-09

**Authors:** Anastasios Apostolos, Maria Drakopoulou, Elias Tolis, Georgios Trantalis, Ioannis Kachrimanidis, Panayotis Vlachakis, Konstantinos Tsioufis, Konstantinos Toutouzas, Konstantina Aggeli

**Affiliations:** ^1^ First Department of Cardiology Medical School, National and Kapodistrian University of Athens, Hippocration General Hospital of Athens Athens Greece; ^2^ Department of Cardiology Harefield Hospital, Royal Brompton and Harefield Hospitals, Guy's and St Thomas' NHS Foundation Trust London UK; ^3^ Faculty of Medicine Imperial College London London UK

**Keywords:** cor triatriatum sinister, cryptogenic stroke, GORE Cardioform Septal Occluder, patent foramen ovale, percutaneous closure, transesophageal echocardiography

## Abstract

We present the case of a 55‐year‐old male with no significant medical history who experienced a cryptogenic ischemic stroke, likely due to a patent foramen ovale (PFO) in the setting of an incomplete cor triatriatum sinister (CTS). Transesophageal echocardiography (TEE) revealed a complex interatrial septum with a long tunnel PFO and an incomplete crescent‐like membrane in the left atrium, consistent with CTS. Despite the anatomical challenges, a 30 mm GORE Cardioform Septal Occluder was successfully implanted. To the best of our knowledge, this is the first case with CTS where PFO closure is performed successfully. This case highlights the technical considerations and feasibility of percutaneous PFO closure in patients with rare congenital anomalies such as incomplete CTS.

## Graphical abstract

We present the case of a 55‐year‐old male who experienced a cryptogenic ischemic stroke, likely due to a patent foramen ovale (PFO) in the setting of an incomplete cor triatriatum sinister (CTS) and a 30 mm GORE Cardioform Septal Occluder was successfully implanted.

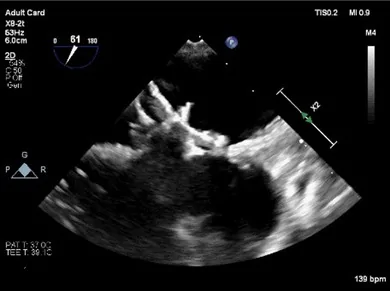

Cor triatriatum is a rare congenital cardiac malformation characterized by the subdivision of an atrium into two separate chambers, affecting either the right or left side of the heart. In cor triatriatum sinister (CTS), the left atrium is typically divided into a proximal (superior) and a distal (inferior) chamber. The proximal chamber receives blood from the pulmonary veins, while the distal chamber communicates with the mitral valve and serves as the functional left atrium. The membrane separating these chambers is thought to result from the incomplete incorporation of the common pulmonary vein into the left atrium during embryonic development (Derimay et al. 2021; Stiermaier et al. 2018). Partial regression of this membrane, without complete obstruction, may result in a fenestrated or incomplete division, which can vary in hemodynamic significance (Kilkenny and Frishman 2023).

In most cases, CTS is hemodynamically insignificant and remains undiagnosed throughout life. However, it can be associated with left atrial outflow obstruction, leading to pulmonary venous congestion and secondary pulmonary hypertension (Fuentes Rojas et al. 2022). In such scenarios, the membrane restricts blood flow from the proximal chamber to the distal chamber, often resulting in symptoms such as dyspnea, recurrent pulmonary infections, or heart failure. Severe cases may present in infancy or early childhood, particularly if the membrane is obstructive, prompting early recognition and intervention (Stiermaier et al. 2018; Hernández‐Benítez and Reyes‐Vázquez 2024; Moya Mur et al. 2014).

Due to its rarity, there is limited experience in managing CTS, which additionally poses significant challenges in the planning and performance of percutaneous cardiac procedures due to its complex morphology. The presence of membranous structures, such as incomplete septation or prominent venous valve remnants, can complicate device‐based interventions, including PFO closure. Herein, we report a case of successful PFO closure in a patient with incomplete CTS using a GORE Cardioform Septal Occluder.

A 55‐year‐old male with no significant past medical history presented with transient vertical diplopia during a transatlantic flight from New York to Athens. After the plane's landing, the patient reported walking weakness; he had a syncope episode of unknown duration, and he was transmitted to the airport health care center where he presented with left hemiplegia, facial droop, and severe dysarthria, which were totally relieved after 45 min.

The patient had a normal body weight (BMI: 26.3 kg/m^2^), was a non‐smoker, exercised regularly, and consumed alcohol socially. His family history was unremarkable, with no reported cardiovascular diseases or sudden cardiac death at an early age.

The patient was transferred to the on‐call hospital and admitted to the Neurology department for further evaluation and management. Initial diagnostic workup included magnetic resonance imaging (MRI), which demonstrated pathological signal intensity in the right thalamic nuclei, with increased T2 and FLAIR signals and restricted diffusion, consistent with acute ischemic changes. Additionally, a few small chronic subcortical microischemic lesions were identified in the frontal lobe. No pathological contrast enhancement was observed. Further investigations, including 48‐h rhythm monitoring, magnetic resonance angiography (MRA), magnetic resonance venography (MRV), triplex ultrasound of the leg veins, and computed tomography angiography of the carotids, yielded unremarkable results. Based on these findings, a diagnosis of cryptogenic stroke was established.

To identify a potential cardioembolic source, transesophageal echocardiography (TEE) was performed. The TEE revealed an incomplete separation of the left atrium by a membrane, consistent with incomplete cor triatriatum sinister (CTS), as well as a long tunnel patent foramen ovale (PFO) with a thick secundum septum (Figure 1). The patient's ROPE score of 7 and Pascal Score of Probable indicated a high likelihood of PFO‐related stroke.

**FIGURE 1:**
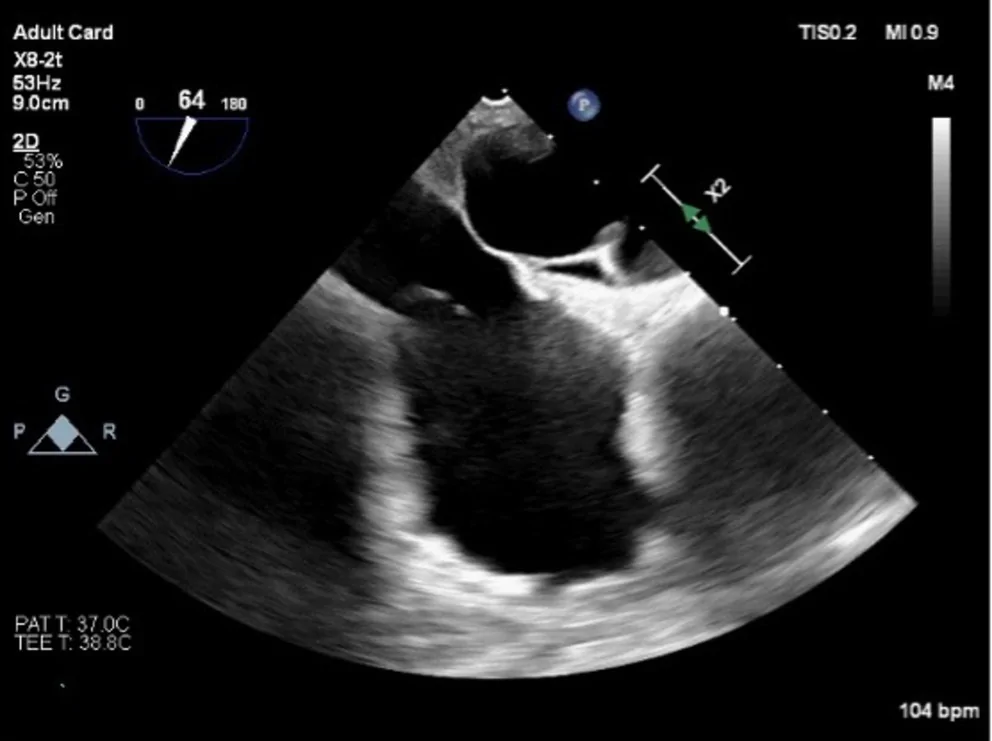
During transoesophageal echocardiography, there was an incomplete crescent‐like membrane at the left atrial septum causing an incomplete separation of the left atrium, findings indicative of Cor triatriatum sinister, and a prominent venous valve remnant such as Eustachian valve.

Despite the anatomical complexity, our Heart Team, in collaboration with neurologists, decided to proceed with percutaneous PFO closure, considering the potential benefit to the patient. Prior PFO closure, coronary angiography was performed and did not reveal any abnormal findings.

A 30 mm GORE Cardioform Septal Occluder was selected due to the thick secundum septum and the long tunnel morphology of the PFO. The procedure was successfully performed, with the incomplete crescent‐like membrane entrapped between the device disks (Figures 2, 3, 4). Post‐procedural bubble testing revealed no residual shunt.

**FIGURE 2:**
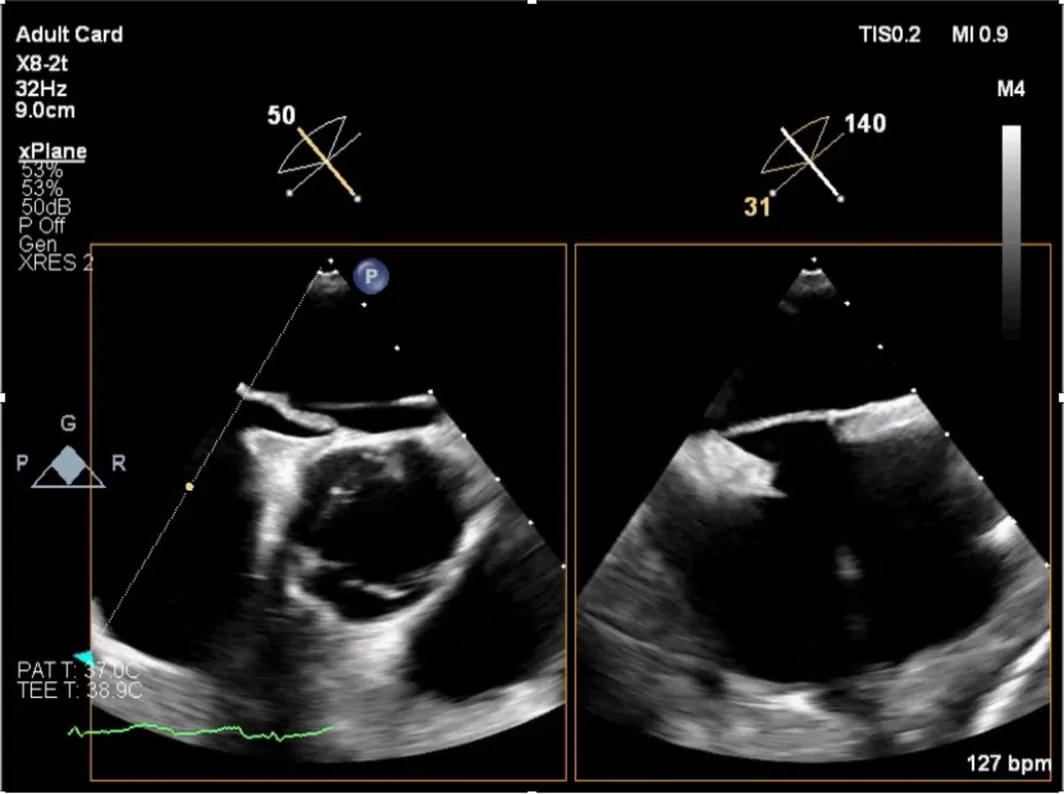
Transesophageal biplane view during passing the wire through patent foramen ovale.

**FIGURE 3:**
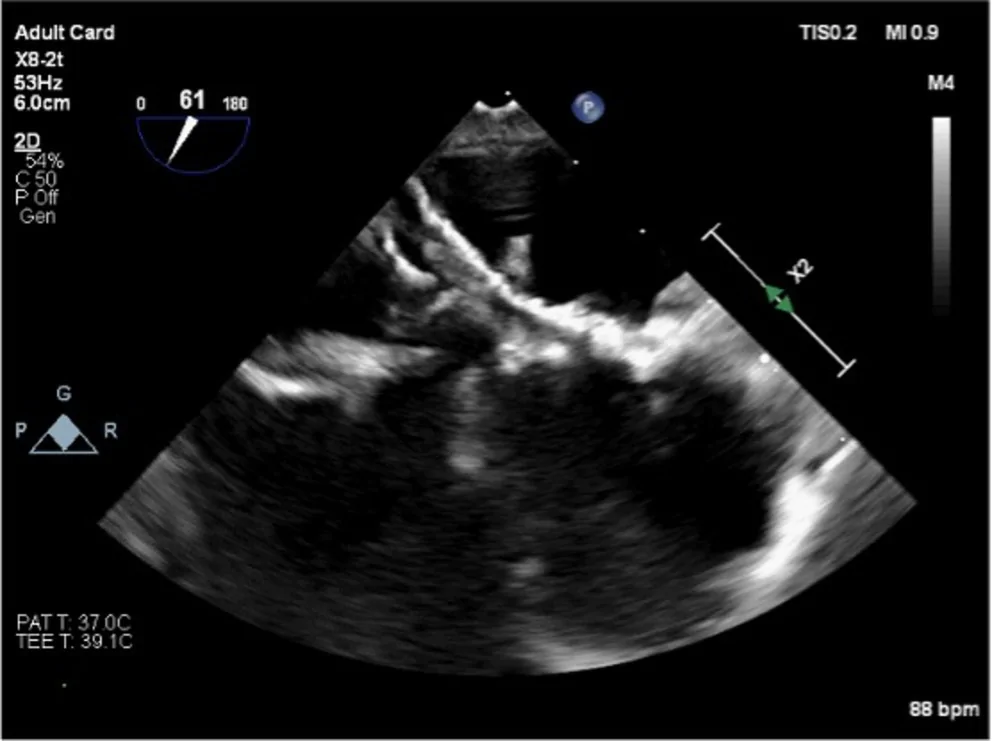
Transoesophageal echocardiography showing the device position after the release.

**FIGURE 4:**
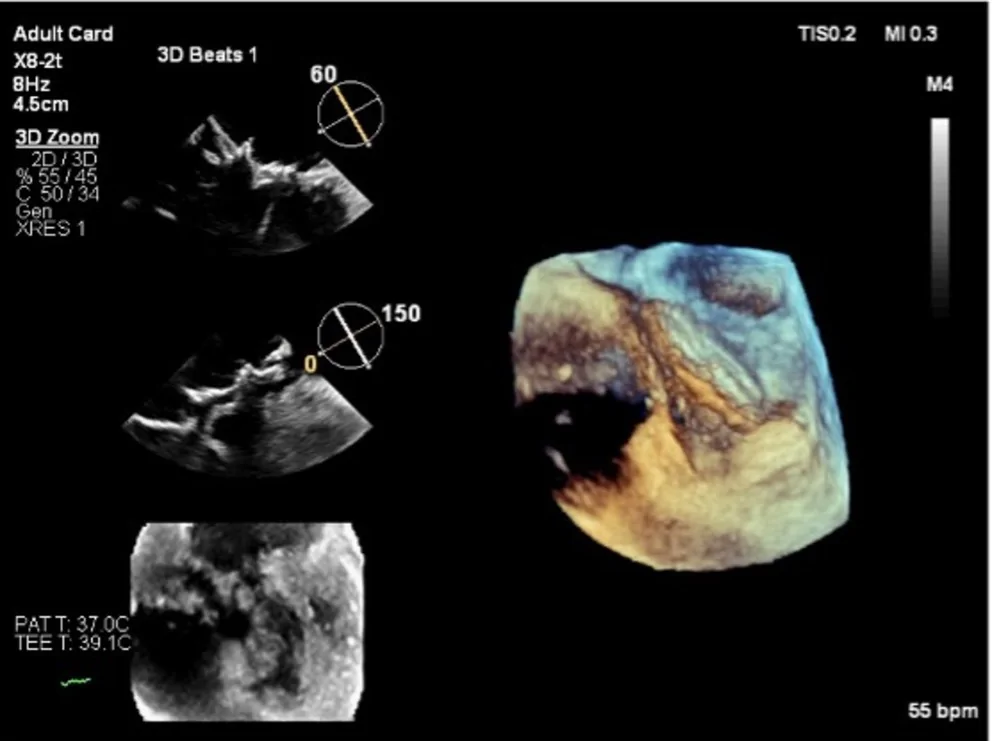
Three‐dimensional transoesophageal echocardiography showing the right disk of the device.

The patient was discharged the day after PFO closure with the following recommendations: dual antiplatelet therapy with aspirin and clopidogrel for 3 months, followed by monotherapy with aspirin for 5 years, atorvastatin 20 mg daily, gastroprotection, and antibiotic prophylaxis for 6 months.

Our case highlights the technical challenges and considerations in managing patients with cryptogenic stroke and complex interatrial anatomy. CTS, though rare, can complicate device‐based closure of a PFO due to the presence of additional membranous structures. To the best of our knowledge, this is the first case report describing the safety and efficacy of PFO closure in CTS anatomy using the GORE Cardioform Septal Occluder.

Transcatheter PFO closure remains the cornerstone of secondary prevention in patients with PFO and cryptogenic stroke (Pristipino et al. 2019; Kavinsky et al. 2022). Although optimal medical therapy with antithrombotic agents provides decent efficacy and adequate safety, transcatheter PFO closure offers more effective secondary prevention of recurrent stroke in selected patients, both in the short and long term (Apostolos et al. 2023). Additionally, it may have a beneficial role in migraine management and decompression illness prevention (Apostolos et al. 2022; Apostolos, Alexiou, et al. 2024; Apostolos, Drakopoulou, et al. 2024). Nevertheless, it should not be considered a panacea, as it has been associated with post‐procedural complications such as atrial fibrillation, device thrombosis, device embolization, and its use in patients with nickel hypersensitivity remains under investigation (Apostolos, Aggeli, and Toutouzas 2024; Apostolos, Tsiachris, et al. 2024).

Although there are no reports describing PFO closure in patients with CTS, a few case reports have demonstrated the safety and efficacy of PFO closure in patients with cor triatriatum dexter (CTD), which involves the right and not left atrium. Recently, David Gomez Martin et al. described three cases involving adult patients (46–53 years old) with CTD and PFO, all presenting with cryptogenic stroke and treated with PFO closure using the Figulla Flex PFO Occluder (Martín et al. 2024). Barca et al. presented a case at the 53rd Congress of the Italian Association of Hospital Cardiologists in Rimini in 2022, involving a 51‐year‐old male patient with a recent diagnosis of paradoxical cardioembolic stroke and PFO, who underwent closure with a multifenestrated Amplatzer septal occluder (18 mm device) (Barca et al. 2022). However, in some studies, PFO closure in patients with CTD was not feasible due to the presence of an immobile membrane angulated toward the interatrial septum (Persia‐Paulino et al. 2022; Maiques Magraner et al. 2019). We preferred the GORE Cardioform Septal Occluder due to its soft and conformable structure, which we considered more suitable for deployment in the setting of complex interatrial anatomy and the presence of an incomplete left atrial membrane.

The presence of an incomplete crescent‐like membrane in the left atrium, a finding with no previously published data in the context of PFO closure, further underscores the need for individualized treatment strategies. The successful entrapment of the membrane between the device disks suggests that percutaneous closure is feasible even in the presence of such anomalies. In such anatomies, PFO closure should only be performed in highly experienced centers, and the device should not be released unless operators are confident of its stable placement.

Percutaneous PFO closure in patients with incomplete CTS is technically feasible and safe; however, it requires careful pre‐procedural planning and device selection. This case demonstrates the successful use of a GORE Cardioform Septal Occluder in a patient with complex interatrial anatomy, providing a potential treatment option for similar cases. Further studies are needed to establish standardized approaches for managing such rare congenital anomalies.

## Funding

The authors have nothing to report.

## Ethics Statement

This case was approved by the ethics committee of the First Department of Cardiology, “Hippocration” General Hospital, School of Medicine, Athens.

## Conflicts of Interest

The authors declare no conflicts of interest.

## Data Availability

The data that support the findings of this study are available from the corresponding author upon reasonable request.
